# A novel, externally validated inflammation-based prognostic algorithm in hepatocellular carcinoma: the prognostic nutritional index (PNI)

**DOI:** 10.1038/bjc.2012.92

**Published:** 2012-03-20

**Authors:** D J Pinato, B V North, R Sharma

**Affiliations:** 1 Division of Experimental Medicine, Imperial College London, Hammersmith Hospital, Du Cane Road, London W12 0HS, UK; 2 Division of Internal Medicine, Universitá degli Studi del Piemonte Orientale, Department of Clinical and Experimental Medicine, Novara, Italy; 3 Statistical Advisory Service, Imperial College London, South Kensington Campus, London, UK

**Keywords:** prognosis, hepatocellular carcinoma, inflammation, prognostic index, survival

## Abstract

**Background:**

There is increasing evidence that the presence of an ongoing systemic inflammatory response is a stage-independent predictor of poor outcome in patients with cancer. The aim of this study was to investigate whether an inflammation-based prognostic score, the prognostic nutritional index (PNI), is associated with overall survival (OS) in patients with hepatocellular carcinoma (HCC).

**Methods:**

All patients with a new diagnosis of HCC presenting to the Medical Oncology Department, Hammersmith Hospital between 1993 and 2011 (*n*=112) were included. Demographic and clinical data were collected. Patients in whom the combined albumin (g l^−1^) × total lymphocyte count × 10^9^ l^−1^ was ⩾45, at presentation, were allocated a PNI score of 0. Patients in whom this total score was <45 were allocated a score of 1. Univariate and multivariate analyses were performed to identify clinicopathological variables associated with OS. Independent predictors of survival identified on multivariate analysis were validated in an independent, stage-matched cohort of 68 patients.

**Results:**

Univariate analyses showed that PNI (*P*=0.003), intrahepatic spread (*P*<0.001), the presence of extrahepatic disease (*P*=0.006), portal vein thrombosis (*P*=0.02), tumour multifocality (*P*=0.003), alfa-fetoprotein >400 ng ml^−1^ (*P*<0.001) and Barcelona Clinic Liver Cancer score (*P*<0.01) were all predictors of OS in the training set. Multivariate analysis revealed the PNI (*P*=0.05), presence of extrahepatic disease (*P*<0.001) and degree of intrahepatic spread (*P*<0.001) as independent predictors of worse OS in this population. The PNI retained independent prognostic value in the validation set (*P*<0.001).

**Conclusion:**

The presence of a systemic inflammatory response, as measured by the PNI, is an independent and externally validated predictor of poor OS in patients with HCC.

Hepatocellular carcinoma (HCC) is the sixth most common neoplasm, and the third most lethal accounting for more than 600 000 deaths per year worldwide (Parkin *et al*, 2005). In countries with active surveillance programs, HCC is diagnosed at an early stage in 30 to 40% of patients and is amenable to potentially curative treatments, such as surgical resection and liver transplantation. However, for the majority of patients palliation is the only treatment option, owing to the fact that most tumors are diagnosed at an advanced stage. Sorafenib is the standard of care in patients with advanced disease, but has limited efficacy, with only a 3-month improvement in overall survival (OS) (Llovet *et al*, 2008). There is a need therefore to further delineate prognostic determinants of survival in order to better stratify those patients likely to benefit from treatment in order to avoid unnecessary toxicity and morbidity.

The pathogenesis of HCC is based on inflammation such that the chronically inflamed liver parenchyma represents a preneoplastic environment in which HCC can arise as a result of the exposure to a plethora of pro-inflammatory stimuli such as infection by hepatotropic viruses, iron or copper accumulation or ethanol consumption (Berasain *et al*, 2009). Unlike other malignancies, survival in HCC patients is peculiarly influenced by the underlying liver function along with the extent of spread of the primary tumour (Bruix *et al*, 2001). The most commonly used prognostic algorithms variably incorporate predictors reflecting liver functional reserve, performance status as well as the tumour stage, however despite there being seven different prognostic scores, there is little consensus in the literature as to the most reliable (Camma and Cabibbo, 2009). The two most commonly used scores are the Barcelona Clinic Liver Cancer (BCLC) (Llovet *et al*, 1999) and Cancer of the Liver Italian Program (CLIP) scores (Investigators CLIP, 1998). The BCLC score is an optimally designed treatment algorithm but recent evidence has shown its limited predictive value in estimating survival in patients with advanced HCC (Huitzil-Melendez *et al*, 2010). Despite having shown good predictive capacity across all the stages of HCC (Hsu *et al*, 2010), the CLIP score subcategorizes patients in seven prognostic strata, therefore reducing its applicability to routine clinical practice. There is a need therefore for a reliable, easy to use prognostic score.

There is increasing evidence showing that the presence of a systemic inflammatory response, as indicated by an elevated circulating C-reactive protein (CRP) concentration, is associated with poor survival in patients with malignancy, including HCC (Hashimoto *et al*, 2005; Nagaoka *et al*, 2007). Furthermore, the presence of an inflammatory response is proposed to be pathogenic in the development of cancer-associated malnutrition (Argiles *et al*, 2003a, 2003b). Nutritional impairment in turn is correlated with poor performance status, shorter survival, and increased mortality in patients with cancer (Andreyev *et al*, 1998; Dewys *et al*, 1980; Rey-Ferro *et al*, 1997; Jagoe *et al*, 2001). This is of particular concern in patients with HCC, given the concomitant underlying illnesses and possible impaired nutritional status secondary to cirrhosis (Meng *et al*, 2010). The prognostic nutritional index (PNI) has been shown to be a prognostic marker in a number of gastrointestinal malignancies (Nozoe *et al*, 2002, 2010; Kanda *et al*, 2011), and, more recently, in a large study by Proctor *et al* (2011), the PNI was found to predict prognosis in malignancy regardless of the site of origin. The aim of the present study is to examine the relationship between the PNI and survival in patients with HCC.

## Patients and methods

We conducted a retrospective analysis of all the patients with a diagnosis of HCC presenting to the Medical Oncology Department, Hammersmith Hospital between 1993 and 2011, excluding those cases with a positive history of inflammatory disease or active concomitant infection. All the patients included satisfied the diagnosis of HCC made according to radiological or histological criteria as recommended by the American Association for the Study of the Liver guidelines (Bruix and Sherman, 2005). Clinical variables, including demographic data, complete blood picture, albumin, alpha-fetoprotein (AFP), staging of the tumour, including the number of focal hepatic lesions, maximum diameter detected during contrast enhancement phase and degree of intra-hepatic spread, and Child–Turcotte–Pugh (CTP) class were collected. The presence of liver cirrhosis was diagnosed using clinical and radiological criteria (computerised tomography scan) in all patients. A proportion of the cirrhotic subjects included in this study had histological evidence of liver cirrhosis (45%). Overall (cancer-specific) survival was computed from the time of referral to our unit.

The PNI was calculated as described previously (Nozoe *et al*, 2010) where combined albumin (g l^−1^) × total lymphocyte count × 10^9^ l^−1^ ⩾45, at presentation, were allocated a PNI score of 0. Patients in whom this total was <45 were allocated a PNI score of 1, where a PNI of 1 is indicative of severe nutritional impairment and PNI of 0 is normal.

The significance of the tested prognostic models was externally validated using an independent set of consecutive, stage-matched patients presenting to the St Mary's and Charing Cross Hospitals (*n*=68).

By using OS as a measure of outcome we further evaluated the independent prognostic power of the PNI in comparison with commonly used prognostic scores, including the CLIP score and the BCLC staging system on the complete set of patients (*n*=180). Both CLIP and BCLC prognostic models were calculated as previously described (Investigators CLIP, 1998; Llovet *et al*, 1999).

Each score was further tested for homogeneity, discrimination and monotonicity in order to estimate the accuracy of outcome prediction as recommended in previously published studies (Ueno *et al*, 2001). The study was approved by the local Research Ethics Committee.

### Statistical analysis

Survival (cancer-specific) analysis was carried out using the Cox proportional hazard model. Multivariate survival analysis was performed using a stepwise backward procedure to derive a final model of the variables that had a significant independent relationship with survival. To remove a variable from the model, the corresponding *P*-value had to be >0.10. To avoid colinearity bias, the independent prognostic power of the PNI was preliminarily tested on a first multivariate model that included the individual variables composing the BCLC and CLIP score.

The discriminative ability of each prognostic score was tested using rms packages of Dr Frank Harrell to identify a subset of predictors by backward elimination (Harrell, 2001). Where we assessed the predictive ability of a Cox proportional hazards model, we compared the actual survival outcomes of usable pairs of patients with the values of their estimated prognostic indices from the Cox model. Where the assessment of prediction of multiple biomarkers was performed, the *C*-index was adjusted within the rms package for the over-optimism produced by modeling and assessment being done on the same data via comparison with 150 bootstrap samples. The monotonicity of each score was assessed with the linear trend *χ*^2^ test, whereas the homogeneity of prognostic prediction across categories was tested using the likelihood ratio test as previously described (Ueno *et al*, 2001). Pearson's *χ*^2^ test was used to test the effects of PNI on clinicopathological factors. All analyses except for *C*-index were performed using SPSS software version 19 (SPSS Inc., Chicago, IL, USA).

## Results

The training set consisted of 112 patients whose baseline clinicopathological characteristics are shown in Table 1. The majority of patients had compensated liver function (Child–Turcotte–Pugh Class A, 65%), had underlying cirrhosis (63%), and were staged as being intermediate (56/112, 50%) or advanced (38/112, 34%) according to the BCLC algorithm. The median age of the patients at study baseline was 65 years (range 20–83). The majority of patients had received at least one line of active treatment (68%), including locoregional (52%) or systemic treatments (21%). All remaining patients were offered best supportive care (32%). At presentation, albumin and total lymphocyte count was measured in all 112 and 105 patients, respectively. Median serum albumin was 32 mg dl^−1^ (range 14–48 mg dl^−1^) and median total lymphocyte count was 1.6 × 10^9^ l^−1^ (0.4–4.4 × 10^9^ l^−1^). Sixty-one patients (55%) had an abnormal PNI.

**Figure 1:**
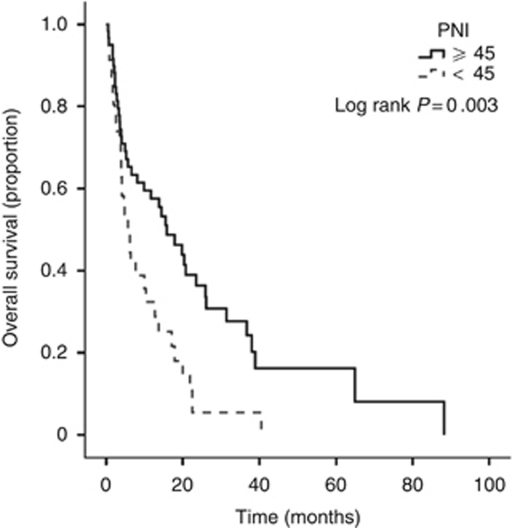
Kaplan–Meier survival curves comparing patients with hepatocellular carcinoma with low-risk PNI score (⩾45) and those with high-risk (<45) PNI score training set).

At the time of analysis 81 (72%) patients had died and overall median survival was 6.4 months (range 1–88 months). On univariate analysis, PNI (*P*<0.05, Figure 1), presence of extrahepatic disease (*P*=0.05), portal vein thrombosis (*P*<0.05), AFP >400 (*P*<0.001), degree of intrahepatic spread (<0.001) and BCLC stage (*P*<0.01) were significant predictors of cancer-specific survival (Table 2). On multivariate analysis, PNI (*P*<0.05), presence of metastases (*P*=0.01) and degree of intrahepatic spread (*P*<0.001) remained significant independent predictors of cancer specific survival. The median survival in patients with a PNI of 0 was 16 compared with 6 months in patients with a PNI of 1 (hazard ratio (HR) 2.02, 95% confidence interval (CI) 1.26–3.23, *P*=0.03).

A significant association was observed between PNI 1 and raised AFP (47%, 20 of 43) (*P*<0.05). Patients with PNI 1 were also more likely to have portal vein thrombosis *P*<0.05) and worse CTP class (*P*<0.001) (Table 1). No other associations were observed.

An independent set of 68 consecutive patients was used to statistically validate the prognostic model identified in the training set. These cases were matching by stage to those in the training set, with the degree of intrahepatic (*P*=0.94) and extrahepatic spread (*P*=0.25), the number of tumours (*P*=0.29) being homogeneously distributed across the groups. Median OS in the validation set was 5.4 months and not significantly different compared with that of the training set (*P*=0.23). In the validation set, the PNI (HR 4.54 95% CI 2.04–10.1, *P*<0.001) together with the presence of extrahepatic disease (HR 3.86 95% CI 1.61–9.22, *P*=0.02) and the extent of intrahepatic spread (HR 1.83 95% CI 1.11–2.99, *P*=0.01) were univariate predictors of survival. The PNI (HR 5.11 95% CI 2.27–11.49, *P*<0.001) and the presence of extrahepatic spread (HR 4.38 95% CI 1.69–11.36, *P*=0.002) were confirmed as independent predictors of OS.

The independent prognostic ability of the PNI, BCLC and CLIP score was then assessed using a multivariate Cox regression model in a combined assessment of all patients (*n*=180). This analysis identified the PNI and CLIP score as the strongest independent predictors of survival (Table 3). As shown in Table 4, the accuracy of the PNI is superior to that of the CLIP and BCLC scores in terms of monotonicity and homogeneity of prognostic prediction, although *C*-index analysis revealed the CLIP score to have superior discriminative ability compared with the other scores.

## Discussion

In the present study, a simple inflammation-based prognostic score (PNI) was shown to be an independent predictor of survival in patients with HCC. This measure of inflammation is based on standard laboratory measurements of total lymphocyte count and albumin, which are routinely measured in the clinical setting. These results are consistent with a number of previous studies investigating the prognostic role of hypoalbuminaemia and lymphocytopenia in both gastro-oesophageal and pancreatic cancer (Nozoe *et al*, 2002, 2010; Kanda *et al*, 2011), as well as liver cirrhosis (Carvalho and Parise, 2006).

The PNI was initially designed to assess the immunological and nutritional aspects of patients undergoing surgery of the gastrointesintal tract, predominantly as an indicator of the nutritional status of any given patient (Nozoe *et al*, 2002, 2010; Kanda *et al*, 2011). Albumin is a widely used indicator of nutrition and has been shown to correlate with post-operative complications (Fujiwara *et al*, 2010; Ellis *et al*, 2011; Lai *et al*, 2011). However there is increasing evidence that the presence of cancer cachexia, partly reflected by a reduction in albumin, is driven by a sustained inflammatory response, either from the tumour itself or as a host reaction (Esper and Harb, 2005). Furthermore, a number of studies have illustrated that the presence of inflammation is reflected in routine haematology with a number of inflammation-based prognostic scores validated in patients with malignancy (McMillan, 2009). In the context of HCC, the neutrophil to lymphocyte ratio (NLR), a simple biomarker derived from the circulating differential white blood cell count, has been associated with reduced survival (Gomez *et al*, 2008; Halazun *et al*, 2009; Chen *et al*, 2011). Another inflammatory index, the modified Glasgow Prognostic Score, based on CRP and albumin has been shown to predict OS in a large cohort of patients (Ishizuka *et al*, 2011). Therefore, although initially thought of as purely a reflection of the nutritional status of a patient, it is likely, given its prognostic association, that the PNI is a reflection of systemic inflammation.

Previous studies have investigated a number of possible prognostic factors in HCC. In particular the adverse effect of portal vein thrombosis, extra-hepatic spread, tumour multifocality and elevated AFP on patients OS has emerged from previously published studies (El-Serag *et al*, 2008). In the present study we found that PNI was superior to the presence of PVT and AFP in predicting OS of a cohort of patients with intermediate-advanced HCC, which is of greater consequence in the management of this patient population, as treatment decisions are often based on the presence of PVT. Consistent with previous studies we found that on multivariate analysis the presence of metastatic disease and intrahepatic spread remained as independent predictors of OS. We noted that PNI correlated significantly with raised AFP, liver functional reserve and the presence of portal vein thrombosis suggesting that a high risk PNI correlates with a more aggressive disease phenotype. Interestingly, the independent prognostic value of the PNI was strengthened by a process of cross-validation, which confirmed the PNI as a stage independent predictor of OS in HCC.

Lastly, our comparative assessment of the PNI with established prognostic systems in HCC confirmed the robustness of the prognostic information conveyed by the PNI, which ranked first in terms of prognostic monotonicity and homogeneity and held a discriminative capacity comparable to that of more complex prognostic systems such as the BCLC. Although the generalizability of our results is limited by the retrospective nature of this clinical study, such finding is of greater consequence in the clinical management of HCC, where there is little consensus in the literature on the optimum staging system, many of which are cumbersome and used only in the clinical trials setting (Camma and Cabibbo, 2009). The population studied consisted of patients presenting to medical oncology outpatient department and thereby consisted predominantly of intermediate and advanced-stage disease. In future studies it would be important to include patients presenting to both hepatobiliary surgeons and hepatologists for routine screening for HCC in order to validate the PNI across all stages of HCC. Moreover, it would be important to compare the utility of PNI with these other staging systems in an independent validation cohort, ideally collected in a prospective fashion.

This step is warranted before the PNI can be used with confidence to estimate survival in individual patients. Furthermore, it would be of interest to correlate PNI with possible pro-inflammatory cytokines and more robust measures of cachexia such as the Patient-Generated Subjective Global Assessment (PGSGA) (Read *et al*, 2005).

This has been previously explored in a small study by Read and colleagues in patients with metastatic colorectal cancer that suggested that there is a correlation with CRP and PGSGA (Read *et al*, 2006). Interestingly a significant association between deranged nutrition scores and inflammatory markers such as low albumin and elevated CRP has been documented in HCC (Tsai *et al*, 2011), suggesting that simple inflammatory markers such as the PNI could be implemented in the routine nutritional assessment.

The mechanism by which systemic inflammation may impact on survival is not completely understood. A number of studies appear to reinforce the biological plausibility behind systemic inflammation and the prognosis of HCC. Lymphopenia is a renown adverse prognostic factor in solid tumours (Ray-Coquard *et al*, 2009) and convincing evidence supports the concept that the impairment of lymphocyte mediated antitumour response is an immunological determinant of patients prognosis in HCC (Yang *et al*, 2010). Moreover, it is well understood that inflammatory pathways can be redirected into a tumour-promoting path by the peritumoural stroma through the activation of innate immunity in HCC (Kuang *et al*, 2011).

This is particularly true in the context of HCC, where cytokines such as interleukin-6 (IL-6) have been identified as candidate molecular risk factors for HCC acting on chronically inflamed hepatocytes (Naugler *et al*, 2007; Prieto, 2008; Falleti *et al*, 2009). In addition, the production of circulating CRP largely upon IL-6 (Kinoshita *et al*, 1999; McKeown *et al*, 2004). Interleukin-6 has been shown to increase the anti-apoptotic and oncogenic potential of tumour cells, as well as inducing drug resistance *in vitro* (Jee *et al*, 2001; Yusuf *et al*, 2003). Furthermore, it has been proposed that elevated CRP identifies those patients with T-lymphocyte impairment, which is associated with poor outcome in malignancy (Canna *et al*, 2005). On the other hand, sustained inflammation may reflect a pro-angiogenic environment, as circulating concentrations of vascular endothelial growth factor are directly associated with CRP, allowing unrestrained tumour growth and dissemination (Kemik *et al*, 2010; Reynes *et al*, 2011).

Hepatic albumin biosynthesis is downregulated by pro-inflammatory stimuli as part of a negative acute phase reaction in patients with malignancy (Steel and Whitehead, 1994). However, impaired synthetic functions accompanying end-stage liver disease needs to be considered as an additional determinant of reduced serum albumin that may have contributed to the results reported. Although previous reports show the independent prognostic value of hypoalbuminaemia in HCC (Hao *et al*, 2009; Nouso *et al*, 2010), our data are not consistent with this finding suggesting that the reduced survival observed in patients with a PNI of 1 was not solely a reflection of impaired liver function.

More recently, the presence of a systemic inflammatory response has been shown to impair the activity of cytochrome 3A (CYP3A4) in patients with advanced cancer patients (Rivory *et al*, 2002; Charles *et al*, 2006). As CYP3A4 is the principal drug-metabolising enzyme for over 60% of all prescribed medications, including sorafenib, changes in the activity of CYP3A4 may result in impaired drug response or increased toxicity (Charles *et al*, 2006). It is further hypothesised that high protein catabolism, and stimulation of the acute phase response, may induce perturbations of the cellular response to chemotherapy-induced DNA damage in normal tissues and result in increased toxicity. Moreover, nutrition may alter the pharmacokinetics of many anticancer agents through altered protein binding and P450 activity (Murry *et al*, 1998; Rivory *et al*, 2002). Prognostic nutritional index therefore, may not only be a valuable tool in prognostication but may also be used to predict those patients at risk of developing toxicity. Dose adjustments before the initiation of treatment in this patient group may improve the tolerability of anticancer agents and help maintain dose intensity. However, this concept requires further evaluation in a prospectively designed trial.

Irrespective of the mechanisms involved, the results of the present study suggest that the presence of a systemic inflammatory response, as indicated by PNI, is a useful tool in the assessment of survival in patients with HCC. As described, the PNI could function as a surrogate marker for the complex interplay between inflammatory pathways, angiogenesis and tumour progression that are known to have an impact on patient survival. PNI is simple to construct from laboratory measures that are routinely assessed in patients before treatment. Prognostic nutritional index therefore should be further evaluated not only as a prognostic marker in patients with HCC at diagnosis, and in the stratification of patients entering clinical trials.

## Figures and Tables

**Table 1:** The relationship between clinicopathological factors and PNI in patients affected by hepatocellular carcinoma (training set, *n*=112)

**Baseline characteristic**	**PNI 0, *N* (%)**	**PNI 1, *N* (%)**	***P***
*Gender*			
Male	94 (86)	49 (79)	
Female	15 (14)	13 (21)	0.3
			
*Aetiology*			
Non-viral	27 (52)	25 (71)	
Viral	25 (48)	10 (29)	0.07^*^
			
*Child–Turcotte–Pugh class*			
A	49 (73)	22 (48)	
B	11 (26)	20 (43)	
C	1 (1)	4 (8)	0.02^*^
			
*Cirrhosis*			
Absent	27 (31)	3 (21)	
Present	60 (69)	11 (79)	0.54
			
*BCLC stage*			
A	10 (16)	6 (13)	
B	35 (57)	19 (42)	
C	15 (25)	17 (38)	
D	1 (2)	3 (7)	0.2
			
*Intrahepatic spread*			
Uninodular <50%	17 (28)	10 (23)	
Multinodular <50%	27 (44)	22 (48)	
Massive ⩾50%	17 (28)	13 (29)	0.8
			
*Number of tumours*			
<3	42 (69)	28 (62)	
⩾3	19 (31)	17 (38)	0.3
			
*Maximum tumour size*			
<5	18 (70)	18 (41)	
⩾5	42 (30)	26 (59)	0.24
			
*Extrahepatic spread*			
Absent	50 (82)	36 (78)	
Present	11 (18)	10 (22)	0.4
			
*AFP (ng ml* ^*−1*^ *)*			
<400	45 (72)	23 (72)	
⩾400	13 (28)	20 (28)	0.01^*^
			
*Portal vein thrombosis*			
Absent	5 (22)	12 (18)	
Present	56 (78)	33 (82)	0.01^*^
			
*AST*			
<40	12 (28)	10 (25)	
>40	31 (72)	29 (75)	0.81
			
*ALT*			
<40	22 (58)	19 (50)	
>40	31 (42)	19 (50)	0.42
			
*ALP*			
<130	22 (40)	15 (35)	
>130	31 (60)	29 (65)	0.56

Abbreviations: AFP=alpha-fetoprotein; ALP=alkaline phosphatase; ALT=alanine aminotransferase; AST=aspartate aminotrasferase; BCLC=Barcelona Clinic Liver Cancer; PNI=prognostic nutritional index.

Associations reaching statistical significance (*P*<0.05) are marked with an asterisk (^*^).

**Table 2:** Univariate and multivariate analysis of prognostic factors of overall survival (Training Set)

**Univariate analysis**				**Multivariate analysis**	
**Variable**	***N*=112**	**Hazard ratio (95% CI)**	***P*-value**	**Hazard ratio (95% CI)**	**P-value**
*Intrahepatic spread*					
Uninodular <50%	28				
Multinodular <50%	52				
Massive⩾50%	31	2.0 (1.4–2.9)	<0.001	2.3 (1.6–3.3)	<0.001
*Number of tumours*					
<3/⩾3	74/53	1.8 (1.2–2.6)	0.003		
*AFP, ng ml^−1^*					
<400/⩾400	71/35	2.7 (1.6–4.4)	<0.001		
*Portal vein thrombosis*					
absent/present	92/18	2.0 (1.1–3.5)	0.02		
*Extrahepatic spread*					
absent/present	90/22	2.0 (1.2–3.5)	0.006	2.8 (1.6–5.0)	<0.001
*PNI*					
0/1	46/61	2.0 (1.3–3.2)	0.003	1.6 (1.0–2.6)	0.05
*BCLC Stage*					
A–B/C–D	73/38	2.1 (1.3–3.3)	<0.01		
*CLIP score*					
0–1/>1	49/62	2.8 (1.7–4.5)	<0.001		

Abbreviations: AFP=alpha-fetoprotein; ALP=alkaline phosphatase; ALT=alanine aminotransferase; AST=aspartate aminotrasferase; BCLC=Barcelona Clinic Liver Cancer score; CI=confidence interval; CLIP=Cancer of the Liver Italian Program; PNI=prognostic nutritional index.

The cutoff values for AFP and intrahepatic spread follow the CLIP prognostic score. Categorization of AST, ALT, ALP was carried out using clinically used cutoff values. To avoid colinearity bias BCLC and CLIP score were not entered into the multivariate model but tested independently as shown in Table 3.

**Table 3:** Multivariate comparison of the prognostic power of PNI, CLIP score and BCLC Stage using a backward stepwise Cox regression model including patients in the training and validation set (*n*=180)

			**95% CI for Exp(*B*)**	
	***P*-value**	**HR**	**Lower**	**Upper**
*Step 1*				
BCLC	0.216	1.091	0.950	1.254
CLIP	0.000	1.373	1.205	1.565
PNI	0.002	1.976	1.286	3.035
				
*Step 2*				
CLIP	0.000	1.418	1.257	1.601
PNI	0.001	2.011	1.308	3.092

Abbreviations: BCLC=Barcelona Clinic Liver Cancer score; CI=confidence interval; CLIP=Cancer of the Liver Italian Program; PNI=prognostic nutritional index.

**Table 4:** Evaluation of prognostic stratification and homogeneity in PNI, CLIP score, and BCLC Stage (*n*=180)

**Prognostic score**	**Linear trend test (chi-square)**	**LR test (chi-square)**	***C*-index (95% CI)**
CLIP	579.728	951.251	0.69 (0.58–0.80)
PNI	840.528	969.102	0.57 (0.48–0.66)
BCLC	701.380	970.338	0.61 (0.51–0.70)

Abbreviations: BCLC=Barcelona Clinic Liver Cancer score; CI=confidence interval; CLIP=Cancer of the Liver Italian Program; PNI=prognostic nutritional index.
